# Survey on the Progression of Myopia in Children and Adolescents in Chongqing During COVID-19 Pandemic

**DOI:** 10.3389/fpubh.2021.646770

**Published:** 2021-04-28

**Authors:** Wujiao Wang, Lu Zhu, Shijie Zheng, Yan Ji, Yongguo Xiang, Bingjing Lv, Liang Xiong, Zhuoyu Li, Shenglan Yi, Hongyun Huang, Li Zhang, Fangli Liu, Wenjuan Wan, Ke Hu

**Affiliations:** ^1^Department of Ophthalmology, The First Affiliated Hospital of Chongqing Medical University, Chongqing Key Laboratory of Ophthalmology and Chongqing Eye Institute, Chongqing, China; ^2^Department of Sports, Health and Arts, Chongqing Municipal Education Commission, Chongqing, China; ^3^Physical, Health and Art Education Research Center, National Institute of Education Sciences, Beijing, China; ^4^Chongqing Medical University, Chongqing, China

**Keywords:** COVID-19, quarantine, myopia, progression, prevention

## Abstract

**Background:** The Covid-19 pandemic restricts children and adolescents from doing normal daily activities such as playing outdoors and going to school. The incidence and prevalence of myopia have increased during the COVID-19 pandemic. The aim of this study was to investigate and evaluate the impact of the home confinement during the COVID-19 pandemic on the progression of myopia among children and adolescents in Chongqing, China.

**Methods:** The survey was conducted by using stratified samplings. Samples were randomly selected from the 2019 National Student Physique and Health Survey database, and their visual function and refractive data were compared with those in 2020. Vision-related behavior questionnaire including digital screen exposure was applied to investigate the correlation between eye parameter and eye health-related behavior.

**Results:** A total of 1,733 and 1,728 students were enrolled in 2020 and 2019, respectively. The percentage of myopia students was 55.02% in 2020, which was higher than that in 2019 (44.62%). The mean uncorrected visual acuity (UCVA, LogMAR, 0.35 ± 0.42) in 2020 was higher than that in 2019 (0.27 ± 0.36, *P* < 0.001). The mean spherical equivalent (SE) refraction (−1.94 ± 2.13 D) in 2020 was lower than that in 2019 (−1.64 ± 5.49 D, *P* < 0.001). For students who used digital devices for online courses, the mean SE in the television group (−1.10 ± 1.49 D) was better than that in the computer group (−2.03 ± 2.37 D, *P* = 0.0017) and in the cell phone group (−2.02 ± 2.09 D, *P* = 0.0028). The average duration of online classes (*r* = −0.27, *P* < 0.0001), the number of online classes per day (*r* = −0.33, *P* < 0.0001), as well as digital screen exposure time (*r* = −0.20, *P* < 0.0001) were negatively correlated with SE, and the average time of outdoor activity (*r* = 0.20, *P* < 0.0001) was positively correlated with SE.

**Conclusions:** Increased digital screen exposure contributes to myopic progression in children and adolescents of Chongqing during the COVID-19 pandemic. Suitable digital devices should be provided for online classes and outdoor activity should be advocated to prevent myopic pandemic.

## Introduction

Since the outbreak of COVID-19 in December 2019, the Chinese government has taken effective measures to fight the pandemic. Home quarantine is one of the main measures at the early stage, which effectively controlled the spread of COVID-19 to a limit district through a series of efforts. The pandemic may have affected people's physical and mental health, especially that of the children and adolescents (1, 2).

Since Asia has a high incidence of myopia (3–7), more attention should be paid to the refractive status of children and adolescents. The Chinese government attaches great importance to the prevention and control of myopia among teenagers. In particular, the impact of the home quarantine during the COVID-19 pandemic on the progression of myopia has become a topic of public concern. In this study, we conducted an epidemiological study to investigate the effect of home confinement during the COVID-19 outbreak on the visual health of young people. We found that the myopia rate was increased after the COVID-19 pandemic, and increased digital screen exposure contributed to myopic progression in young people. This research is important for the scientific assessment of how the COVID-19 pandemic influenced the visual function of children and adolescents. The use of appropriate digital devices may be important in preventing and controlling myopia among children and adolescents in the “post-COVID-19 period.”

## Methods

### Subjects

The Ethics Board of the First Affiliated Hospital of Chongqing Medical University approved this study. All procedures were performed in accordance with the tenets of the Declaration of Helsinki.

This study included students of grades 1–6 in primary school, grades 1 and 2 in junior school, and grades 1 and 2 in high school. The students were randomly selected from three districts in Chongqing, including the Shapingba District, the Dazu District, and the Yunyang District. One class was randomly selected from each grade in each school, and all students in the class were selected in the study. If the class had <25 students, this insufficiency was resolved by drawing from neighboring classes, resulting in no <25 students in each class.

The students were from in four rural schools, including 618 students in 2019 and in 2020, and seven urban schools, including 1,110 students in 2019 and 1,115 students in 2020. Information of the 1,728 students (860 males and 868 females) and 1,733 students (860 males and 873 females) were collected in 2019 and 2020, respectively.

### Research Design

All the data in 2019 were obtained from the database of the 2019 National Student Physique and Health Survey, which was a national census held in October 2019, with assessments of the height, weight, and visual function of the students. The data in 2020 were collected from the same samples, with loss of information on five students in 2020. The survey was conducted using stratified samplings. The sampling method met the national sampling requirements and had sufficient sample size.

### Visual Acuity Measurement and Questionnaire

To investigate the association between the lifestyle and visual status, all students were required to accomplish the visual inspection and questionnaires about the genetic and environment issues that might cause myopia. The questionnaire mainly included information about changes in visual function after the pandemic, outdoor activity time, online class times, and types of digital devices. The questionnaires were filled out by the students themselves who were in and above the fourth grade or completed under with parents' help.

The visual acuity and refractive status in the 2019 National Student Physique and Health Survey were monitored and tracked. The 2020 National Student Physique and Health Survey was used to determine the alterations in the refractive status of the same samples before and after the pandemic using information in the 2019 National Student Physique and Health Survey as the control. Visual acuity examination and questionnaire survey were conducted from June 17 to June 24 in 2020.

The mean uncorrected visual acuity (UCVA) was monocularly assessed and recorded in LogMAR scores by using the standard logarithmic visual acuity chart. The mean spherical equivalent (SE) refraction was recorded by an optometry unit (Supore, China). Myopia was defined as mean UCVA < 5.0 with mean SE refraction < −0.50 D (8). SE was converted by diopter of spherical power (DS) plus 1/2 diopter of cylindrical power (DC). The average progression rate of myopia is represented by diopter change value/diopter value of 2020. The increment over 1.0 D was defined as rapid myopic progression, while <0.25 D was slow progression (9). To avoid bias, only the right eye was examined.

### Statistical Analysis

Data analysis was performed using GraphPad Prism 8 (GraphPad Software, Inc.). Data with normal distribution are presented as the mean ± standard deviation, and data without normal distribution are presented as median (*M*) and interquartile range (P25 and P75). Count data are presented as frequency (rate). The sample size and other influencing factors were corrected by binary logistic regression. Logistic regression was used to investigate the influencing factors of SE progression. The Mann–Whitney test and Kruskal–Wallis test were used to compare the differences among variables. The Wilcoxon matched pair test was used to compare differences between paired variables. Categorical variables were analyzed using Pearson's chi-square test or Fisher's exact test. Pearson's and Spearman's tests were applied for correlation analysis. *P*-values lower than 0.05 were considered statistically significant.

## Results

### General Information

A total of 1,728 students in 2019 and 1,733 students in 2020 were included in the study. There were no significant differences in region, gender, or grade among the samples. The distribution of schools and population is listed in Table 1.

**Table 1:** General information of the survey.

**Categories**	**2019**				**2020**			
	**Students (*n*)**	**Percentage (%)**	***X*^**2**^**	***P***	**Students (*n*)**	**Percentage (%)**	***X*^**2**^**	***P***
Rural	618	35.76	0.0022	0.9618	618	35.66	0.0047	0.9451
Urban	1,110	64.24			1,115	64.34		
Male	860	49.77	0.0020	0.9641	860	49.62	0.0053	0.9419
Female	868	50.23			873	50.38		
Primary school	1,045	60.47	0.0030	0.9985	1,045	60.30	0.0092	0.9954
Junior school	353	20.43			357	20.60		
High school	330	19.10			331	19.10		
Total	1,728	100.00	N/A	N/A	1,733	100.00	N/A	N/A

### SE Progression and Influencing Factors

A total of 36 and 93 students were included in the rapid myopia group and slow myopia group, respectively. The average progression rate of myopia for this sample set was 10.49%, and the average SE progression rate in the slow myopia group (0.04 ± 0.28 D) was lower than that in the rapid myopia group (0.68 ± 0.26 D). Logistic regression analysis was performed to compare the behavior differences between the rapid myopic progression group and slow myopic increment group with genetic factors, the average time of online courses per day, and outdoor activity time. The average time of online courses (*r* = −0.37, *P* = 0.1112) and the outdoor activity time (*r* = −0.06, *P* = 0.7978) were negatively correlated with SE progression. Genetic factors was positively correlated with SE progression (*r* = 1.20, *P* = 0.0064).

### Higher Myopia Rate Among Teenagers in Different Grades After the Pandemic

**Figure 1:**
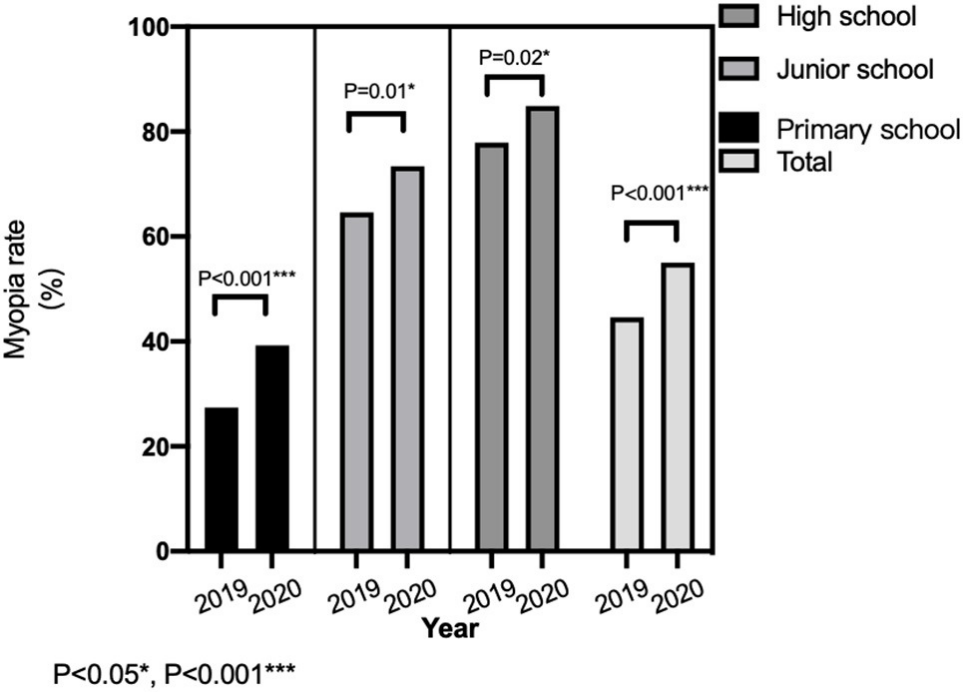
Comparison of myopia rate among teenagers in different age groups before and after the epidemic.

The overall percentage of myopia among teenagers in 2020 was 55.02%, which was increased by 10.40% as compared to that in 2019 (44.62%). In 2020, the percentages of myopia were 84.89% in high school, 73.39% in junior school, and 39.27% in primary school, which were significantly higher than those in 2019 (*P* < 0.05; Figure 1).

### Visual Acuity and Refractive Status Before and After the Pandemic

The mean UCVA (0.27 ± 0.36) in 2019 was lower than that in 2020 (0.35 ± 0.42, *P* < 0.0001). The SE (−1.64 ± 5.49 D) in 2019 was higher than that in 2020 (−1.94 ± 2.13, *P* < 0.001).

### Visual Acuity and Refractive Status Between Different Genders

In 2019, the UCVA (LogMAR) scores were 0.26 ± 0.36 in males and 0.27 ± 0.36 in females. There were no significant differences in the UCVA between males and females (*P* = 0.9661). The mean UCVA in males (0.34 ± 0.43) was not significantly different from that in females in 2020 (0.36 ± 0.42, *P* = 0.5809). In addition, there was no difference in the mean SE between males and females in 2019 and in 2020 (*P* > 0.05).

### Genetic Factors

To address the genetic factors on myopic progression, the refractive status of the students' parents were collected in 2020. The UCVA in myopic parents was higher than that in emmetropic parents (*P* < 0.0001). The SE was lower in myopic parents than in emmetropic parents (*P* = 0.001; Table 2).

**Table 2:** The mean uncorrected visual acuity and spherical equivalent refraction with myopic/emmetropic parents of 2020.

	**Students (*n*)**	**Mean UCVA ± SD**	**Mean SE(D) ± SD**
Students with myopic parents	621	0.42 ± 0.43	−2.27 ± 2.26
Students with emmetropic parents	1,112	0.31 ± 0.41	−1.76 ± 2.04
*p*		<0.0001	0.001

*UCVA, uncorrected visual acuity; SE, spherical equivalent refraction; SD, standard deviation*.

### Regional Distribution

**Figure 2:**
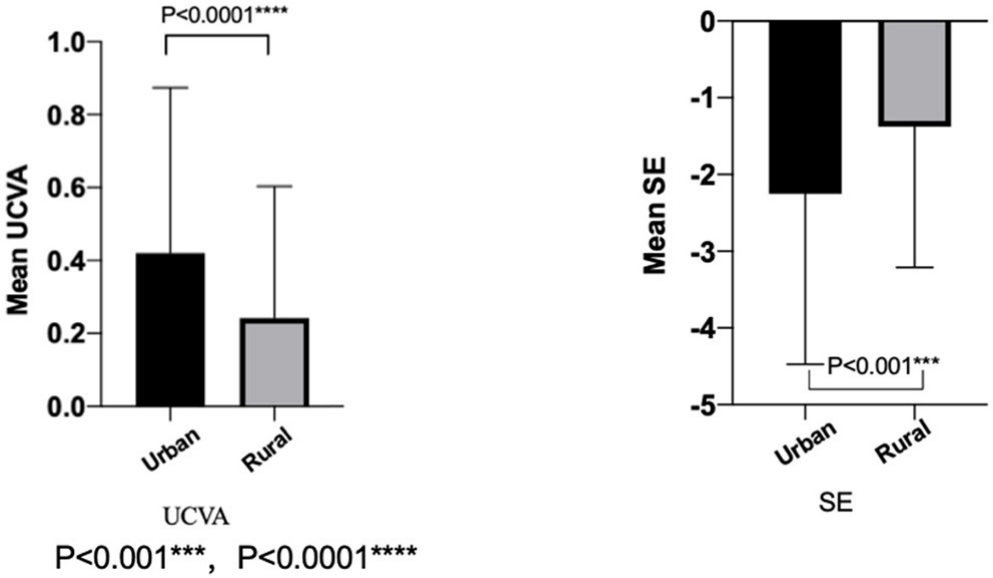
The mean uncorrected visual acuity and spherical equivalent refraction in the urban and the rural of 2020.

The UCVA (LogMAR) was 0.24 ± 0.36 in rural students, which was significantly better than that in urban students (0.42 ± 0.45, *P* < 0.0001). The SE in urban students (−2.25 ± 2.22 D) was lower than the SE in rural students (−1.38 ± 1.83 D, *P* < 0.001; Figure 2).

### Digital Device Exposure

**Figure 3:**
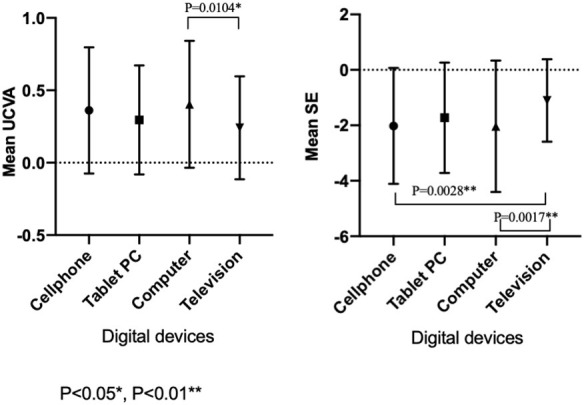
The mean uncorrected visual acuity and spherical equivalent refraction with different digital devices used for education in 2020.

Among all the students recruited in 2020, uses of cell phones, tablet PC, computers, televisions, and projectors for online classes were 992 (57.2%), 310 (17.9%), 391 (22.6%), 36 (2.1%), and 3 (0.2%), respectively. The usage of projectors was too small to perform a statistical comparison. The UCVA (LogMAR; *P* = 0.0037) and the SE (*P* = 0.0009) were significantly different among the students who use cell phones, tablet PC, computers, and televisions (Table 3). The UCVA (LogMAR) in the television group (0.24 ± 0.35) was lower than that in the computer group (0.40 ± 0.43, *P* = 0.0104). The SE in the television group (−1.10 ± 1.49 D) was significantly better than that in the computer group (−2.03 ± 2.37 D, *P* = 0.0017) and in the cell phone group (−2.02 ± 2.09 D, *P* = 0.0028; Figure 3).

**Table 3:** The mean uncorrected visual acuity and spherical equivalent refraction with different digital devices used for online classes during home confinement period.

**Digital device**	**Students (*n*)**	**Mean UCVA ± SD**	**Mean SE(D) ± SD**
Cellphone	992 (57.2%)	0.36 ± 0.43	−2.02 ± 2.09
Tablet PC	310 (17.9%)	0.29 ± 0.37	−1.73 ± 1.99
Computer	391 (22.6%)	0.40 ± 0.43	−2.03 ± 2.37
Television	36 (2.1%)	0.24 ± 0.35	−1.10 ± 1.49
Projector	2 (0.2%)	N/A	N/A
*p*		0.0037	0.0009

*UCVA, uncorrected visual acuity; SE, spherical equivalent refraction; SD, standard deviation*.

### Correlation Analysis

Correlation analysis was performed to determine the association of SE with outdoor activity time, digital screen time, and the number and average time of online courses per day. The average time of online courses (*r* = −0.27), the number of online courses per day (*r* = −0.33), and screen time (*r* = −0.20) were negatively correlated with SE (*P* < 0.0001). Outdoor activity time (*r* = 0.20) was positively correlated with SE (*P* < 0.0001).

## Discussion

In this survey on the progression of myopia in children and adolescents in Chongqing in 2019 and 2020, we demonstrated that the myopic rate increased in 2020 after the COVID-19 pandemic compared with that in 2019 before the pandemic. Hereditary factors, regional differences, digital screen exposure time, and the types of digital devices used for online classes, as well as the time spent for outdoor activities, were associated with myopic progression during the COVID-19 outbreak. Our findings suggest that the pandemic has accelerated the progress of myopia in children and adolescents.

The home quarantine measure has been proven to be an effective way to control the COVID-19 pandemic (10). However, it also led to less outdoor activity time and more online classes for teenagers. The percentage of myopia among children and adolescents in 2020 was increased by 10.40% as compared to that in 2019 in Chongqing. Similarly, the myopic rate was increased by 11.7% in a previous survey on the refractive conditions in nine provinces in China (11). Sumitha et al. (12) reported that approximately 79% of 917 students were myopic between March and April in 2020 in India.

In terms of regional differences, the 2020 myopia survey showed that students from rural areas outperformed urban students on both mean UCVA and SE. These results are consistent with the previous study (13), showing that students in urban regions spent less outdoor time than those in rural regions, and urbanized students spend more time watching TV rather than have outdoor exposure (14). In addition, it has been demonstrated that children with high myopic parents have a high risk of high myopia (15–17), which is consistent with our findings.

In this study, we found that outdoor activity was closely associated with myopic progression. It has been reported that, compared with those in Western countries, students in certain Asian countries who undergo more academic pressure had higher incidence of myopia and spent less time doing outdoor activities and physical exercises (18, 19). Effective outdoor promotion activities can reduce the onset of myopia (20, 21). Home quarantine in the early stage of the COVID-19 outbreak reduced the outdoor activity time for children and adolescents, which might have further contributed to the increased myopic rate. Therefore, we suggest that although self-protection is critical, outdoor activities should be appropriately performed to prevent myopia during the COVID-19 pandemic.

Digital screen exposure time as well as the digital devices used for online studies might also produce an effect on myopic progression in teenagers. According to our survey, most students mainly used mobile phones and computers for online learning. However, they had worse UCVA and SE than those who used TV for studying. Over the past few years, cell phones and tablet PC have become popular in younger generations (one in three children aged 1–6 years use phones for 1–2 h per day) (22, 23). Cross-sectional and cohort studies have shown that the use of computers is closely associated with the prevalence of myopia and increased myopic refractive error in children of 5–16 years (24–29). During the COVID-19 pandemic, home confinement and school closure resulted in more online learning in order to maintain the normal process of academic tasks. Recent studies have revealed that long-term education and academic tasks that expose people to electronic products increase myopia (7, 30, 31). Longer digital screen exposure time can cause a higher risk of myopia (7, 32). Our results also demonstrated that excessive use of digital screens, especially with the device closer to the eye, is more likely to induce myopic development. The World Health Organization (WHO) offers a guideline to limit sedentary screen time for kids under 5 years old (33). Hence, parents should ask their kids to keep a safe distance while reading in online classes or to adopt alternative products like TV or projectors with relatively longer reading distance.

This survey collected comparable data from Chongqing in detail in order to compare Chinese students' refractive problems before and after the pandemic, which might provide important evidences for public health (34–36). School, along with family education and parental instructions, is important for regular eye care for children and adolescents to prevent myopic development. The government should also consider educational policies, improve optometric services, and offer affordable corrective lenses for the huge need of refractive correction (37). Meanwhile, progressive addition of executive bifocal spectacle lenses, peripheral defocusing lenses, and overnight orthokeratology have already been proposed for myopia retardation (38).

### Limitations

The SE in the television group was better than that in the computer group and in the cell phone group, but the sample size of the television group was much smaller than the other two groups, so we will further conduct a prospective study and expand the sample size to verify with valid statistical analysis whether the conclusions will be the same.

## Conclusions

Due to the COVID-19 pandemic, in China, the teaching mode was transferred from school to family and from offline to online. We found that the percentage of myopic students increased by 10.40% during this period in Chongqing, China. Decreased outdoor activity time, increased digital screen exposure time, and the types of digital devices used for online studies were related to the myopic progression. Therefore, it is urgent for the government and schools to take measures such as resuming regular classes when the pandemic is under control. To alleviate the negative impact on myopia development, numerous outdoor activities should be advocated and appropriate digital devices should be provided for online classes.

## Data Availability Statement

The original contributions presented in the study are included in the article/Supplementary Material, further inquiries can be directed to the corresponding author/s.

## Ethics Statement

The studies involving human participants were reviewed and approved by the Ethics Board of the First Affiliated Hospital of Chongqing Medical University. Written informed consent to participate in this study was provided by the participants' legal guardian/next of kin. Consent for publication has been obtained.

## Author Contributions

WuW, SZ, and WeW processed and analyzed the data. LuZ, YX, BL, LX, ZL, and SY provided constructive advice for conception and data analysis. WuW analyzed the data and wrote the manuscript. KH and WeW designed the study and reviewed the manuscript. KH and HH supervised the study. All authors contributed to the article and approved the submitted version.

## Conflict of Interest

The authors declare that the research was conducted in the absence of any commercial or financial relationships that could be construed as a potential conflict of interest.
