# Supplementary material for: Survey on the Progression of Myopia in Children and Adolescents in Chongqing During COVID-19 Pandemic
Source: Front Public Health. 2021 Apr 28;9:646770. doi: 10.3389/fpubh.2021.646770 (PMC8115404; doi:10.3389/fpubh.2021.646770)
Supplement: Supplementary file 1 [file Table_1.DOCX]

Visual Acuity Behavior Questionnaire

| Questionnaire on visual acuity - related behavior of primary and middle school students | | |
| --- | --- | --- |
| Province (city/autonomous Region) : □ City (state) : □ Region: □ (Economic status 1; 2; 3) Monitoring point: □ (1 city; 2 suburb) school name (seal) : __ _ _ date of filling: year: month: day: students name: __________________ | | |
| Number | Contents of survey | Choices |
| A、Basic information |  |  |
| A01 | Grade | □□ |
| A02 | Identity code 4 bits | □□□□ |
| A03 | Gender | 1. Male 2. Female |
| A04 | Birth date | □□□□year□□month□□day |
| A05 | Is your father myopic | 1. Yes 2. No |
| A06 | Is your mother myopic | 1. Yes 2. No |
| B、Myopic related symptoms |  |  |
| B01 | In the last 5 months, do you feel that your eyesight has lost | 1. Yes 2. No 3 unclear |
| B02 | Have your eyes become tired or dry in the last 5 months | 1. Yes 2. No  3. Unclear |
| B03 | At the beginning of January this year, were you myopic | 1. Yes 2. No |
| B04 | In early January, were you wearing glasses or not | 1. Yes  2. If no, please jump to B05 |
| B041 | If you already wear glasses, your glasses strength | Degree of your right eye  degree of your right eye |
| B05 | If it is June, are you myopic | 1. Yes 2. No |
| B06 | Now in June, do you wear glasses or not | 1. Yes  2. If no, please jump to C01 |
| B061 | If you already wear glasses, your glasses strength | Degree of your right eye□□□□  Degree of your left eye□□□□ |
| C、Behavior with the eye |  |  |
| C01 | Do you need online classes every day (computer, tablet or phone) | 1. Yes 2. No |
| C02 | What tools do you usually use to go to school online courses? (Most used) | 1. Computers 2. tablet PCs 3. Phones 4. Television 5. Projector 6.Otherwise, please state ____ |
| C03 | The total amount of time you spend online each day | 1. Less than 1 hour (including no online class) 2. 1-2.5 (excluding 2.5) hours 3. 2.5-4 (excluding 4) hours 4. At least 4 hours   5. unknown |
| C04 | You need to take several online classes every day | Average ___ online course |
| C05 | The time of each online class is approximately | Average ___ minutes |
| C06 | Is there any break in the middle of each online class | 1. Yes 2. No |
| C07 | Do you look far away when you are resting | 1. Yes 2. No |
| C08 | How much time have you spent watching TV, computer, mobile phone and tablet (including surfing the Internet, watching movies, surfing the Web and playing games) every day for the past 5 months? | 1. Less than 1 hour 2. 1-2.5 (excluding 2.5) hours 3. 2.5-4 (excluding 4) hours 4. 4 and above 5. unknown |
| C09 | The amount of time you played video games every day for the past 5 months | The average___ hours ___minutes every day |
| C10 | Have you read a book or watched a video (tablet or phone) while lying down in the last 5 months | 1. Never 2. Sometimes 3. Often |
| C11 | Do you continue to look at your phone or tablet after turning off the lights in the last 5 months | 1. Never 2. Sometimes 3. Often |
| C12 | Did your parents or teacher remind you that your reading and writing posture is incorrect? | 1. Never 2. Sometimes 3. Often |
| D、Environment |  |  |
| D01 | Whether there are windows on the left side of desks to let the sunshine in | 1. Yes 2. No 3. Unknown |
| D02 | Whether your desk bright enough for study on a sunny day | 1. Very bright, no need to turn on lamp or roof lamp 2. Generally bright, need to turn on lamp 3. Very dark, no lamp 4.Unknown |
| D03 | What light do you use for studying at night | 1. Use both a lamp and a roof lamp  2. Use a lamp 3. Use a roof lamp 4. Other ________ |
| D04 | Do you think the lights are bright enough to study at night | 1. Yes 2. No 3. Unknown |
| D05 | Do you think the height of your study desk and chair is suitable for your height | 1. It is too tall and needs to be bent over. 2. 2. It is too short and needs padding. 3. Suitable well 4. Unknown |
| E、Outdoor activities and physical exercise |  |  |
| E01 | In the last seven days, how many days did you engage in vigorous physical activity, such as lifting weights, digging, aerobic exercise, or fast cycling? | □ ____day(s) a week □ Skip to question E03 without related physical activity |
| E02 | How much time do you normally spend on vigorous physical activity each day during the 7 days? | □ __hour(s)___minute(s)___every day  □ Unknown or not sure |
| E03 | How many days in the last seven have you been moderately physically active, such as lifting light items, cycling at a normal pace or playing tennis in pairs? Please don't include walking. | □ __days a week □ No moderate physical activity jumps to question E05 |
| E04 | During this period, how much time do you usually spend on moderate physical activity each day? | □ ____hour(s)____minute(s) a day  □ Unknown or not sure |
| E05 | How many of the last 7 days have you walked, and walked for at least 10 minutes at a time? | □ ___day(s) a week □ If no walking please jump to problem E07 |
| E06 | During that time, how much time do you usually spend walking? | □ ____hour(s)____minute(s) a day □ Unknown or not sure |
| E07 | How much of the workday have you been sitting in the last 7 days? | □ ___hour(s)____minute(s)  □ Unknown or not sure |
| E08 | How much sun exposure have you had per day in the past week? (Time spent in direct sunlight) | 1. Less than 1 hour   2. 1-2 (excluding 2) hours  3. 2-3 (excluding 3) hours  4. 3 and above  5. Unknown |
| F、Eating behavior and others |  |  |
| F01 | How many times in the past 7 days have you eaten sweets (including candy, cake, chocolate, sweet soup, etc.) | 1. Never 2. Less than once a day  3. At least once a day |
| F02 | How many times in the past 7 days have you had a sugary drink (e.g., Coke, iced tea, crushed orange, Nutrition Express) | 1. Never 2. Less than once a day  3. At least once a day |
| F03 | How many times have you eaten Fried food (such as deep-fried dough sticks, oil cakes, French fries, Fried chicken wings, etc.) in the past 7 days | 1. Never 2. Less than once a day  3. Ar least once a day |
| F04 | How long do you sleep a day | □ ____hour(s)____minute(s) a day □ Unknown or not sure |
| F05 | Are you sleeping well | 1. Good 2. General, occasionally wake up in the middle of the night can not sleep 3. Bad, often wake up in the middle of the night can not sleep 4. Unclear |
| F06 | Do you still feel tired in the morning | 1. Yes 2. No 3. Unclear |
